# Disorders of Deglutition

**Published:** 1923-10

**Authors:** J. P. I. Harty

**Affiliations:** Honorary Surgeon to the Ear, Nose and Throat Department, Bristol Royal Infirmary


### DISORDERS OF DEGLUTITION.

BY

J. P. I. Harty, B.A., M.B., B.Ch., F.R.C.S.,

*Honorary Surgeon to the Ear, Nose and Throat Department, Bristol Royal Infirmary.*

---

Before considering the disorders of deglutition it is well to recall certain normal processes involved in this function.

*In the laryngo-pharynx* the most important mechanism is the closure of the larynx. This is effected by the apposition and tilting forward of the arytenoids and the apposition of the aryepiglottic folds, while a raising of the larynx brings the arytenoids with their cornicula into contact with the cushion of the epiglottis. The contractions in sequence of the middle and inferior constrictors of the pharynx cause the bolus to glide in turn over the posterior surfaces of the epiglottis and the arytenoids and to enter the hypo-pharynx.

According to Chevalier Jackson the bolus is split into two as it passes over the epiglottis, each half passing into the corresponding pyriform sinus and from there into the hypo-pharynx. The hypo-pharynx, or post-cricoid region, is the narrowest part of the pharynx, and its walls are kept in tight apposition by the tonic action of the crico-pharyngeus muscle. The crico-pharyngeus muscle is the specialised lower transverse circular portion of the inferior constrictor. Attached in front to the lateral margins of the signet portion of the cricoid cartilage, it is fixed behind to the median raphe of the pharynx. A large percentage of ingested foreign bodies become impacted in this region, and it is the point where difficulty in introducing the œsophagoscope is experienced, firm, steady pressure having to be exerted to overcome the tonic action of the crico-pharyngeus.

*In the œsophagus* the food is definitely passed along by peristaltic action which is under nervous control. These waves can be seen and actually felt in the horse, and are easily demonstrable by skiagraphy with Barium paste in the human subject.

As viewed through the œsophagoscope the upper portion of the thoracic œsophagus is definitely a patent tube, exhibiting clearly respiratory and cardiac movements. The lumen is seen to increase during inspiration and diminish during expiration, while the cardiac movements are transmitted from the adjacent thoracic aorta.

Owing to the thoracic œsophagus not pursuing a straight course the patency of the lumen cannot be seen in its full extent. Starting slightly to the left of the middle line, it then assumes a median position, and finally again inclines to the left; but besides this it follows the curve of the thoracic spine, and finally proceeds fairly abruptly forward to pass through the diaphragm. It is important to remember these deviations from the straight line in passing the œsophagoscope. The abdominal portion of the œsophagus is one inch in length.

With regard to the presence of a sphincter at the cardiac orifice much dispute reigns. According to Chevalier Jackson no true sphincter is present, but apposition is maintained at the diaphragmatic aperture by the tonic "pinch cock" action of fibres from the crura of the diaphragm, and further that on retrograde œsophagoscopy the cardia and abdominal œsophagus do not seem to exist. I have not had an opportunity to verify this, but there is no doubt that the tendency is, "Where an aperture exists, there shall a sphincter be described."

The presence of a deglutition centre in the medulla controlling this important function is probable, though not proven.

The method I propose to adopt in this paper is to take in sequence the different subdivisions of the digestive tube involved in deglutition, and discuss the disorders in deglutition produced by disease in that particular area.

For purposes of classification I am grouping these into (1) Mural, where the wall of the tube is involved; (2) Extra-mural. Those of nervous origin whether central or peripheral are best treated separately.

It is important to remember that "dysphagia," or difficulty in swallowing, includes three conditions: (1) Obstructive difficulty; (2) painful difficulty; (3) paralytic difficulty.

In taking the history care should be taken to elucidate which of these three conditions exists or predominates.

**The mouth.**—I. *Deformities and Defects.* (*a*) Congenital: Cleft palate. The difficulties in nutrition in these cases both from the sucking and swallowing point of view are too well known to require any further allusion. Macroglossia. (*b*) Acquired: (1) Perforation of the palate, usually syphilitic in origin; (2) dental defects (insufficient mastication); (3) complete removal of tongue—gurgling swallowing, the head having to be thrown back. II. *Inflammatory.* When the floor of the mouth is involved the movements of the tongue and also the mylohyoid muscle are hampered, and so the initiation of deglutition interfered with. The dribbling of saliva in these cases is a marked sign, and a great discomfort to the patient. III. *Syphilitic lesions,* except, perhaps, a gumma of the tongue, do not cause interference with deglutition. IV. *Tuberculous and lupus* lesions are comparatively rare, and do not interfere with deglutition. V. *Neoplasms,* except from their actual size, do not influence deglutition ; but carcinoma when involving the floor of the mouth gives rise to profuse salivation and painful deglutition. VI. *Myasthenia gravis*, a muscular disease affecting deglutition, will operate here as well as upon the subsequent subdivisons of the digestive tube. VII. *Extra-mural.* (1) Fractures of the lower jaw ; (2) wounds, etc.

**The oro-pharynx. A. Mural.** — I. *Defects and Deformities.* None of these produce any noticeable effect on deglutition. II. *Inflammatory.* Pharyngitis, tonsillitis, Vincent's angina, etc. The effects of all of these on swallowing are well known, while the painful deglutition and altered speech in peritonsillar abscess are characteristic. III. *Syphilitic lesions* produce a certain amount of discomfort, but no real interference with swallowing. If severe, they may subsequently lead to a certain amount of pharyngeal stenosis. IV. *Tuberculous and lupus lesions.* Tuberculous lesions in this region are not common except in the tonsil, and that has no effect on deglutition. *Lupus* of the posterior pharyngeal wall is usually associated with lupus of the nose, naso-pharynx and larynx : it does not interfere with deglutition. V. *Neoplasms*, unless from their size, do not produce any serious difficulty in swallowing. Malignant growths with ulceration are associated with painful deglutition. (*a*) *Benign.* I have seen the following associated with some symptoms : Aberrant thyroid at the base of tongue ; cysts at the base of tongue ; hairy mole involving palate and fauces ; ossification of stylohyoid ligament in tonsil ; enlarged tonsils. (*b*) *Malignant.* Epithelioma of tonsil, pharyngeal wall, or base of tongue. Sarcoma of tonsil. VI. *Foreign bodies.* It is very rare for foreign bodies, unless of exceptionally large size, to become impacted here ; but fish-bones buried in the tonsil are fairly common, and give rise to painful deglutition. VII. *Myasthenia gravis* will also operate here.

**B. Extra-mural.**—(1) Retro-pharyngeal abscess; (2) neoplasms of vertebral column; (3) aneurism (internal carotid); (4) fibro-sarcoma of naso-pharynx; (5) large simple choanal polypus.

**In the laryngo-pharynx. Mural.**—I. *Deformities and Defects. Imperforate pharynx.* In these cases the pharynx ends blindly, and either the upper end of the œsophagus may be represented by a fibrous cord attached to the pharynx, or the œsophagus may have no connection with the pharynx, and open into the trachea as high as the third ring or even into the left bronchus. II. *Inflammatory.* Streptococcal ulceration may occur here; but as a rule any effects on deglutition are masked by the dyspnœa dependent on associated œdema of the larynx. III. *Syphilitic.* Ulcers or gummata of the posterior pharyngeal wall or vestibule of the larynx may produce some discomfort in deglutition, but the effects on respiration and the voice are more prominent. IV. *Tuberculous and Lupus.* Tuberculous lesions of the larynx produce painful deglutition, especially when situated on the vestibule of the larynx or the posterior surfaces of the arytenoids. Painful deglutition may be the most marked symptom, and later actual obstruction may occur. The diagnosis from malignant disease is often most difficult. Lupus in this region may proceed to almost complete destruction of the epiglottis (a favourite site) without any effect on deglutition beyond a slight sense of discomfort. V. *Neoplasms* are practically all laryngeal. *Benign* produce respiratory and not deglutition symptoms. *Malignant.* Epitheliomais very common. Sarcoma rare in this region. The favourite site of epithelioma is the posterior surface of one or other arytenoid, less frequently one of the pyriform fossæ. The lateral wall of the pharynx and the vestibule of the larynx are subsequently involved through extension. The growth occurs much more frequently in men than in women. Owing to its position painful deglutition is an early and very important symptom, respiratory and voice disturbances occur later, and subsequently actual obstruction to deglutition is superimposed. An early involvement of the lymphatic glands always occurs. This is in marked contradistinction to epithelioma of the vocal cords (intrinsic), where glandular involvement is very rare. VI. *Foreign bodies,* unless very large, either pass through this region to be held up in the hypo-pharynx, or are impacted in the larynx, producing respiratory symptoms. VII. *Myasthenia gravis* also affects the muscles of this subdivision of the digestive tube.

**Hypo-pharynx.**—I. *Foreign bodies.* The great majority of ingested foreign bodies are impacted either here or at the level of the superior aperture of the thorax. I have removed lumps of meat, fish and meat bones, coins, rings and other trinkets from this region. When opaque to X-rays they are seen on skiagraphy to lie in the coronal plane, as against the sagittal plane in the larynx or trachea. It is important to remember that an impacted foreign body does not as a rule cause complete obstruction to deglutition. II. *Pharyngeal diverticulum.* This is a pulsion hernial protrusion of the mucous membrane, which appears between the lower oblique fibres and the transverse or crico-pharyngeus portion of the inferior constrictor muscle of the pharynx. It commences posteriorly in the middle line, but as a rule then deviates to one or the other side, usually the left. They are often associated with œsophageal obstruction as an exciting cause. The fundus of the sac may obstruct the œsophagus through pressure lower down in the neck. The main symptom is the return of undigested food some time after a meal. A swelling in the neck can usually be felt, and pressure on this determines the return of food. A certain amount of obstructive difficulty is usually present. Skiagraphy with barium paste will definitely show the presence of a diverticulum. III. *Epithelioma* in this region is quite common, and is practically confined to women. I have seen it in women under 30 years of age, and as is usual in carcinoma appearing elsewhere at an early age, it is very rapid in its growth and fatal termination. Obstructive difficulty is the symptom in these cases. On examination by the indirect method with the laryngeal mirror the growth is invisible until a late and advanced stage, when it has risen out of the hypo-pharynx. The only suspicious sign to be seen by the laryngeal mirror in the early stage is an increase of mucus and saliva at the entrance to the hypo-pharynx and in the pyriform fossæ. Examination by the direct method is the only means of making an early diagnosis, combined with the removal of a portion for pathological investigation. X-ray screening with barium paste or capsules is in my experience unreliable in this region.

**In the œsophagus.**—**A. Mural.** I. *Deformities and Defects.* Congenital strictures and webbing. Imperforate œsophagus. Cicatricial strictures following the swallowing of corrosive fluids. The sites in order of frequency are: (*a*) crossing of left bronchus (level); (*b*) hypo-pharynx; (*c*) diaphragmatic opening. II. *Inflammatory*, starting from the trauma of a passing foreign body. III. *Syphilis.* This condition is rare, but gumma, ulceration or the resultant fibrosis with stricture may be found. IV. *Tuberculosis.* This is rare, but it occurs from infection by swallowed sputum; it is usually terminal. V. *Neoplasms.* Benign are rare. Mucous polypus, papilloma, fibroma, angioma, varices, and aberrant thyroid have been recorded. *Malignant.* Sarcoma is rare, and Epithelioma is fairly common, occurring in both sexes; the usual sites in order of frequency are: (1) Middle third, more prevalent in women; (2) Lower third, more prevalent in men. VI. *Foreign bodies.* The usual sites of impaction are : (1) At the level of the thoracic entrance ; (2) at the level of the aortic narrowing ; (3) at the level of the left bronchus. VII. *Myasthenia gravis* may possibly act here.

**B. Extra-mural.**—It is necessary here to consider separately the three subdivisions of the œsophagus. *Cervical :* Carcinoma of thyroid ; cysts and simple tumours of thyroid ; pharyngeal diverticulum ; enlarged glands ; dislocation backwards of the sternal end of clavicle. *Thoracic :* Aneurism ; mediastinal growths ; enlarged glands ; pericarditis. *Abdominal :* Enlargement of the left lobe of the liver.

**Of nervous origin.**—These lesions are naturally grouped into the two main types—organic and functional, the former being subdivided into central and peripheral. **A. Organic.**—I. *Central in origin :* Bulbar paralysis ; syringomyelia ; disseminated sclerosis ; Landry's paralysis ; tabes (rare). In all of these the whole of the digestive tube from the mouth to the cardia is affected, and both sensory and motor fibres are involved. II. *Peripheral.* Post-diphtheritic paralysis. In these cases the muscles of the palate and pharynx are those usually involved, but the œsophagus may sometimes be implicated. The same results are occasionally seen after scarlet fever and streptococcal infections. Facial paralysis only produces difficulty in mastication. In my experience unilateral paralysis of the ninth, tenth, eleventh and twelfth cranial nerves has no appreciable effect on deglutition (*e.g.* Trotter's syndrome). **B. Functional.**—Functional disorders may affect any of the previously mentioned subdivisions of the digestive tube. (1) Inability to start the bolus on its way from the mouth (unable to take pills, "small swallow," etc.). (2) Inability to engage the bolus by the muscles of the isthmus faucium and to contract the pharyngeal constrictors. This is associated with the symptom of "lump rising in the throat." (3) Sense of obstruction in the hypo-pharynx due to lack of relaxation of the crico-pharyngeus muscle at the correct moment or to its over-action. (4) Paresis of œsophageal muscle.

A word must here be said about the condition commonly known as cardio-spasm. If the presence of a sphincter be established, then the term explains itself as a spasm of that sphincter. Chevalier Jackson maintains that it is not a spasm but a flaw in the reflex relaxation of the "pinch cock" crural fibres at the diaphragmatic aperture. In some of these cases marked dilatation of the œsophagus ("œsophagocele") occurs above the obstruction.

The deglutition disorders which occur in rabies and occasionally tetanus are probably of toxic central origin.

The sensory neuroses of the pharynx deserve mention here. It is my experience that in most functional cases hypo-æsthesia is combined with paræsthesia, so that the examination of the pharynx and larynx is particularly easy. Hyper-æsthesia is very evident in alcoholic cases, and the laryngologist has the truth of the perverted proverb, "The wages of gin is breath," forcibly brought home to him. Attempts at concealment by musk in women and onions in men do not improve the situation.

To summarise with a few general statements. *Painful difficulty* ceases at the level of the œsophagus, and is, as a rule, accurately located by the patient. Three conditions should be thought of: (1) Foreign body; (2) tuberculous larynx; (3) epithelioma of the larynx. In the first the history of ingestion of the foreign body is always given by the patient. To eliminate the second careful examination of the chest supplemented by X-rays and inquiry as to hæmoptysis is essential. Conditions of the mouth and oral-pharynx are easily visible. *Obstructive difficulty*, when situated in the œsophagus, is referred by the patient to one or other end; obstruction above this level by the sufferer is as a rule accurately located.

Early diagnosis is essential. An operable case soon becomes inoperable and hopeless. The policy should be "Look and see," not "Wait and see."

### Reviews of Books.

